# Chronic Kidney Disease-Induced Cardiac Fibrosis Is Ameliorated by Reducing Circulating Levels of a Non-Dialysable Uremic Toxin, Indoxyl Sulfate

**DOI:** 10.1371/journal.pone.0041281

**Published:** 2012-07-19

**Authors:** Suree Lekawanvijit, Andrew R. Kompa, Minako Manabe, Bing H. Wang, Robyn G. Langham, Fuyuhiko Nishijima, Darren J. Kelly, Henry Krum

**Affiliations:** 1 Centre of Cardiovascular Research and Education in Therapeutics, Department of Epidemiology and Preventive Medicine, Monash University, Melbourne, Australia; 2 Department of Medicine, University of Melbourne, St. Vincent’s Hospital, Melbourne, Australia; 3 Pharmaceutical Department, Kureha Corporation, Tokyo, Japan; 4 Department of Nephrology, St. Vincent’s Hospital, Melbourne, Australia; Virginia Commonwealth University, United States of America

## Abstract

Cardiovascular death commonly occurs in patients with chronic kidney disease. Indoxyl sulfate (IS), a uremic toxin, has been demonstrated *in vitro* as a contributory factor in cardiac fibrosis, a typical pathological finding in uremic cardiomyopathy. This study aimed to determine if cardiac fibrosis is reversible by lowering serum IS levels using an oral charcoal adsorbent, AST-120. Subtotal-nephrectomized (5/6-STNx) Sprague-Dawley rats were randomized to receive either AST-120 (AST-120, n = 13) or no treatment (vehicle, n = 17) for 12 weeks. Sham operated rats (n = 12) were used as controls. Early left ventricular (LV) diastolic dysfunction was demonstrated by an increase in peak velocity of atrial filling [A and A’ waves] and a decrease of E/A and E’/A’ ratios obtained by echocardiography. This was accompanied by a 4.5-fold increase in serum IS (p<0.001) as well as elevated tail-cuff blood pressure (p<0.001) and heart weight (p<0.001). Increased LV fibrosis (p<0.001), gene expression of pro-fibrotic (TGF-β, CTGF) and hypertrophic (ANP, β-MHC and α-skeletal muscle actin) markers, as well as TGF-β and phosphorylated NF-κB protein expression were observed in STNx + vehicle rats. Treatment with AST-120 reduced serum creatinine (by 54%, p<0.05) and urine total protein (by 27%, p<0.05) vs vehicle whilst having no effect on blood pressure (AST-120 = 227±11 vs vehicle  = 224±8 mmHg, ns) and heart weight. The increase in serum IS was prevented with AST-120 (by 100%, p<0.001) which was accompanied by reduced LV fibrosis (68%, p<0.01) and TGF-β and phosphorylated NF-κB protein expression (back to sham levels, p<0.05) despite no significant change in LV function. In conclusion, STNx resulted in increased cardiac fibrosis and circulating IS levels. Reduction of IS with AST-120 normalizes cardiac fibrosis, in a blood pressure independent manner.

## Introduction

Chronic kidney disease (CKD) is a major contributor to cardiovascular (CV) mortality which is responsible for 40–50% of all deaths in such patients. [1] Death from cardiac causes is greater in dialyzed uremic patients by a factor of approximately 10–30 compared to the rates in the respective background population. [2].

Studies on cardiac pathology in CKD have previously demonstrated typical structural changes, “uremic cardiomyopathy”, comprising fibrosis, hypertrophy and a reduction in capillary supply per unit volume of cardiac tissue. [3] Renal fibrosis is also a key characteristic finding in progressive CKD, irrespective of the nature of the initial renal insult.

A variety of mechanisms contributing to uremic cardiomyopathy have been proposed such as neurohormonal activation specifically that of the renin-angiotensin-aldosterone and sympathetic nervous systems, [4], [5] hemodynamic alterations (especially hypertension), [6] anemia, [6], [7] pro-inflammatory cytokine activation, [8] and oxidative stress. [9] Treatments targeting these pathways have demonstrated beneficial effects on these structural and functional changes as well as major clinical outcomes. [10], [11], [12], [13].

An important pathophysiological finding generally overlooked as being contributory to cardiac dysfunction in CKD is the accumulation of uremic toxins which are normally excreted by the healthy kidney. In renal failure patients on conventional hemodialysis, most of the accumulated toxins can be removed, however some are insufficiently due to high protein-binding. Indoxyl sulfate (IS) is one such protein-bound, poorly-dialysable uremic toxin. IS is an intestinal bacterial metabolite derived from dietary tryptophan, [14] approximately 90% of circulating IS is albumin-bound. [15] A pro-fibrotic effect of IS on the kidney has been reported and found to be associated with CKD progression. [16] Treatment with an IS-reducing agent, AST-120, has been shown to reverse kidney fibrosis and delay CKD progression by adsorbing indole (the IS precursor) in the gut. [16], [17].

We have recently demonstrated that IS has both pro-fibrotic and pro-hypertrophic effects on cardiac cells *in vitro*. [18] These findings therefore suggest that IS may be implicated in uremic cardiomyopathy. Therefore, the aim of the present *in vivo* study was to test this hypothesis by determining whether IS-reducing therapy may have beneficial effects on the cardiac manifestations of CKD.

## Results

The final total number of animals used in this study was 42 (Sham = 12, STNx + Vehicle  = 17, STNx + AST-120 = 13). The mortality rate after STNx surgery was 64.29% (54/84), this was not different between the two STNx groups (61.36% for STNx + Vehicle vs 67.5% for STNx + AST-120, p = 0.22).

### Animal Characteristics

Table 1 shows general characteristics and renal function parameters. A significant decrease in body weight (BW) was observed in STNx + vehicle animals. The systolic tail-cuff BP was significantly increased at 4, 8 and 12 weeks post-surgery in both STNx groups.

**Table 1:** Animal characteristics and renal function assessment.

	Sham	STNx + Vehicle	STNx + AST-120
Body weight (g)	522.7±14.8	469.2±13.2*	489.9±14.5
Kidney/BW (g/kg)	6.3±0.2	4.7±0.2***	4.2±0.1***
SBP (mmHg)			
Week 4	141±5	210±6***	208±7***
Week 8	142±4	206±7***	227±10***
Week 12	144±3	224±8***	227±11***
Serum creatinine (µmol/L)	40.25±3.98	115.20±12.03***	74.58±6.98*** ^,^ †
Urine total protein (mg/day)	52.62±4.97	340.4±22.03***	263.5±27.91*** ^,^ †
GFR (mL/min/kg)	10.93±0.35	0.93±0.22***	1.31±0.29***
Creatinine clearance (mL/min)	3.47±0.34	1.19±0.15***	1.54±0.15***
Hemoglobin (g/L)	151.4±1.66	121.9±2.88***	123.9±4.87***
Serum IS (mg/dL)			
Baseline (week 0)	0.17±0.02	0.15±0.02	0.17±0.01
Week 8	0.17±0.02	0.71±0.06***	0.15±0.01†††
Week 12	0.18±0.02	0.83±0.08***	0.18±0.03†††
Urine IS (mg/dL)			
Week 8	24.36±2.03	10.10±0.69***	3.00±0.42†††
Week 12	30.29±2.16	11.17±0.78***	2.13±0.20†††

Data are presented as means ± SEM.

* p<0.05.

*** p<0.001 vs Sham.

† p<0.05.

††† p<0.001 vs STNx + Vehicle.

SBP, systolic tail-cuff blood pressure; GFR, glomerular filtration rate; IS, indoxyl sulfate.

### Renal Function Assessment

Compared to sham, STNx animals had significantly higher serum creatinine and urine total protein levels, and reduced GFR/kg, creatinine clearance and hemoglobin levels. Treatment with AST-120 significantly reduced serum creatinine by 54.2% and urine total protein by 26.7% whilst having no effect on blood pressure (Table 1).

### Indoxyl Sulfate Levels

At week 8 and 12, STNx + Vehicle animals significantly had a higher serum IS level than sham animals. A reduction in serum IS, back to sham levels, was found in STNx + AST-120 animals from 8 weeks post-treatment. Baseline serum IS levels were comparable among the groups (Table 1).

Serum IS at endpoint (12 weeks) showed a significant positive correlation with serum creatinine (p<0.0001, r^2^ = 0.6268) and 24-hour urine total protein (p = 0.03, r^2^ = 0.1573); expressed a significant negative correlation with creatinine clearance (p = 0.0006, r^2^ = 0.3714) and GFR (p = 0.0006, r^2^ = 0.3500).

Urine IS was significantly reduced in STNx + Vehicle animals compared to sham animals and was significantly lower in STNx + AST-120 than STNx + Vehicle animals (Table 1).

### Cardiac Function and Hemodynamic Assessment

The heart weight/body weight (HW/BW), left ventricular weight/body weight (LV/BW) and lung weight/body weight were significantly increased in STNx + Vehicle and STNx + AST-120 compared to sham animals at 12 weeks. There was no difference between STNx + Vehicle and STNx + AST-120 animals (Table 2).

**Table 2:** Cardiac function assessment.

	Sham (n = 12)	STNx + Vehicle (n = 17)	STNx + AST-120 (n = 13)
HW/BW (g/kg)	2.6±0.1	3.9±0.2***	3.7±0.2***
LV/BW (g/kg)	1.8±0.1	3.0±0.1***	2.8±0.1***
Lung weight/BW (g/kg)	3.1±0.1	3.6±0.1**	3.4±0.1**
Echocardiography			
LV mass (g/m^2^)	1.55±0.03	1.98±0.06***	2.00±0.08***
LVPWd (mm)	1.63±0.03	2.09±0.12**	2.06±0.11**
IVSd (mm)	1.66±0.05	2.03±0.09**	2.05±0.12**
E/A ratio	1.91±0.15	1.43±0.09*	1.55±0.15**
A velocity (m/s)	0.52±0.03	0.74±0.05**	0.74±0.06**
E’/A’ ratio	1.62±0.20	1.11±0.11*	1.13±0.12*
A’ velocity (cm/s)	2.60±0.20	3.46±0.24*	4.44±0.667*
E/E’ ratio	0.25±0.01	0.30±0.02^p = 0.08^	0.29±0.03
Millar catheterization			
τ Logistic (msec)	9.43±0.61	13.55±1.03*	12.84±0.67
τ Weiss-in steady state (msec)	12.07±0.53	15.22±0.81*	14.28±0.78
LVESP (mmHg)	86.95±3.87	86.37±6.04	92.82±9.34
dP/dt_max_ (mmHg/s)	5258±539	4837±350	4989±385
PRSW (mmHg)	76.12±7.10	69.92±3.88	76.60±5.03
LVEDP (mmHg)	5.26±0.49	6.42±0.47	6.21±0.40
dP/dt_min_ (mmHg/s)	−4813±428	−3940±337	−4483±480

Data are presented as means ± SEM.

* p<0.05.

*** p<0.001 vs Sham.

† p<0.05.

††† p<0.001 vs STNx + Vehicle.

HW/BW - heart weight/body weight; LV/BW - left ventricular weight/body weight; LV mass - left ventricular mass; IVSd - interventricular septal thickness in diastole; LVPWd - LV posterior wall thickness in diastole; dP/dt_max_ - rate of LV pressure rise; dP/dt_min_ - rate of LV pressure fall; LVEDP - LV end diastolic pressure; LVESP - LV end systolic pressure; PRSW - preload recruitable stroke work; τ (Tau) - load independent measure of isovolumetric relaxation time.

#### Echocardiographic study

Compared to sham, both STNx groups developed early diastolic dysfunction and cardiac hypertrophy.

E/A ratio and E′/A′ ratio was significantly reduced; and A velocity and A′ velocity was significantly increased in STNx + Vehicle and STNx + AST-120, however no difference between these two groups was observed (Table 2). A trend toward increased E/E′ ratio was observed in STNx + vehicle animals (p = 0.08).

LV mass, and individual interventricular septal (IVSd) and posterior wall thicknesses (LVPWd) in diastole were significantly greater in STNx + Vehicle and STNx + AST-120 with no difference between these two groups.

#### Hemodynamic assessment

Pressure-volume loop analysis showed a significant prolongation of τ Logistic and τ Weiss following IVC occlusion and in steady state respectively in STNx + Vehicle; this was not reversed by AST-120 treatment (Table 2). Left ventricular end diastolic pressure (LVEDP), dP/dt_min_ (mmHg/s), left ventricular end systolic pressure (LVESP), dP/dt_max_ (mmHg/s) and the slope of the preload recruitable stroke work (PRSW) relationship were not different among the three groups (Table 2).

### Cardiac Tissue Studies

#### Interstitial matrix deposition

**Figure 1:**
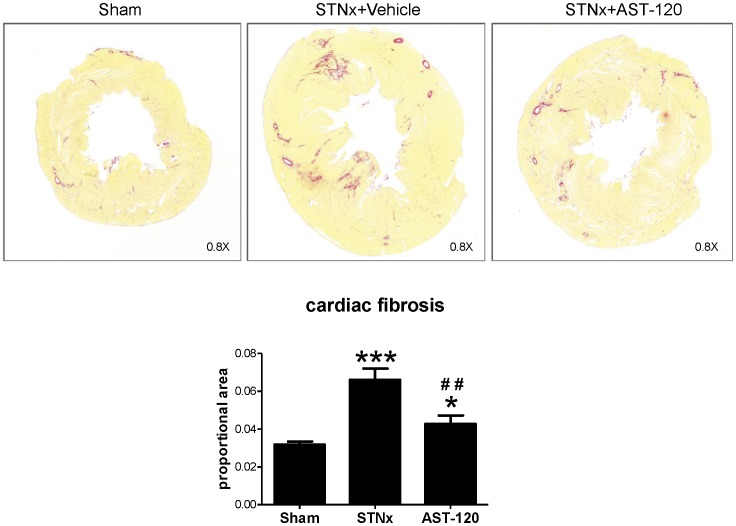
AST-120 reduces cardiac fibrosis in the STNx model. Representative LV images (Picrosirius red staining, magnification, X0.8) show patchy fibrosis, stained in red, in STNx + vehicle which is significantly increased compared to sham. Treatment with AST-120 significantly reduces cardiac fibrosis in the STNx model. *p<0.05, ***p<0.001 vs Sham; ^##^p<0.01 vs STNx + vehicle.

Cardiac interstitial fibrosis was significantly increased two-fold in STNx + Vehicle compared to sham animals (p<0.0001). STNx + AST-120 significantly reduced cardiac interstitial fibrosis by 68% (p<0.01 vs STNx + Vehicle) (Fig. 1).

**Figure 2:**
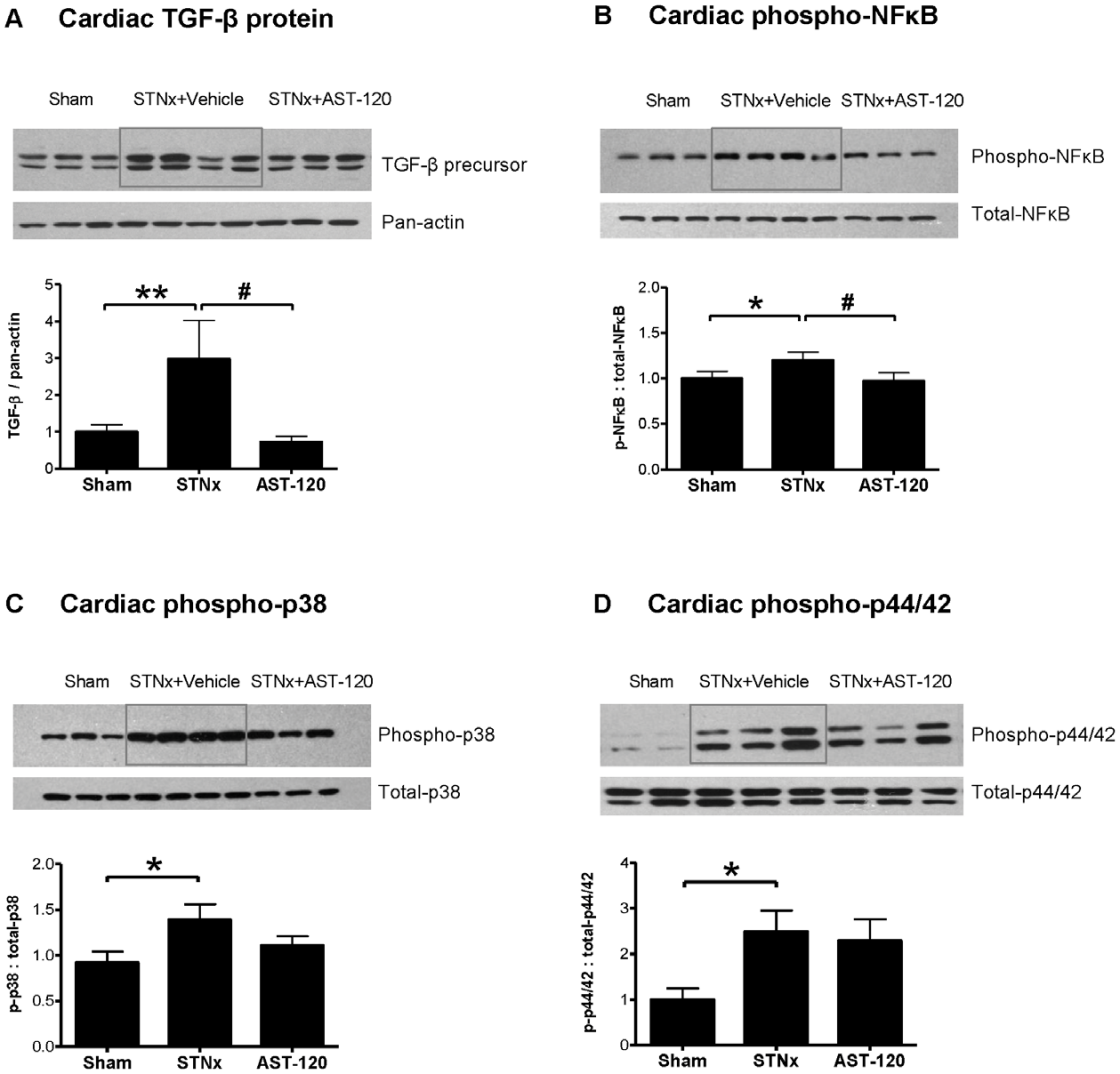
AST-120 normalises cardiac TGF-β and phospho-NFκB expression in the STNx model. Compared to sham, STNx animals show an increase in cardiac TGF-β, phospho-NFκB, phospho-p38 and phospho-p44/42 expression (A, B, C, and D, respectively). AST-120 significantly suppresses cardiac TGF-β and phospho-NFκB (A and B) but not phospho-p38 and phospho-p44/42 expression (C and D). *p<0.05, **p<0.01 vs Sham; ^#^p<0.05 vs STNx + vehicle (NB: All gel images are presented exactly as originally captured except phospho-44/42 which was rearranged into the same order as the others.).

Western blot analysis demonstrated a significant increase in TGF-β, phospho-NFκB, phospho-p38 and phospho-p44/42, protein expression in STNx + Vehicle (Fig. 2). Following treatment with AST-120, a significant reduction in TGF-β and phospho-NFκB was observed in STNx + Vehicle (p<0.05) back to sham levels (Fig. 2).

**Figure 3:**
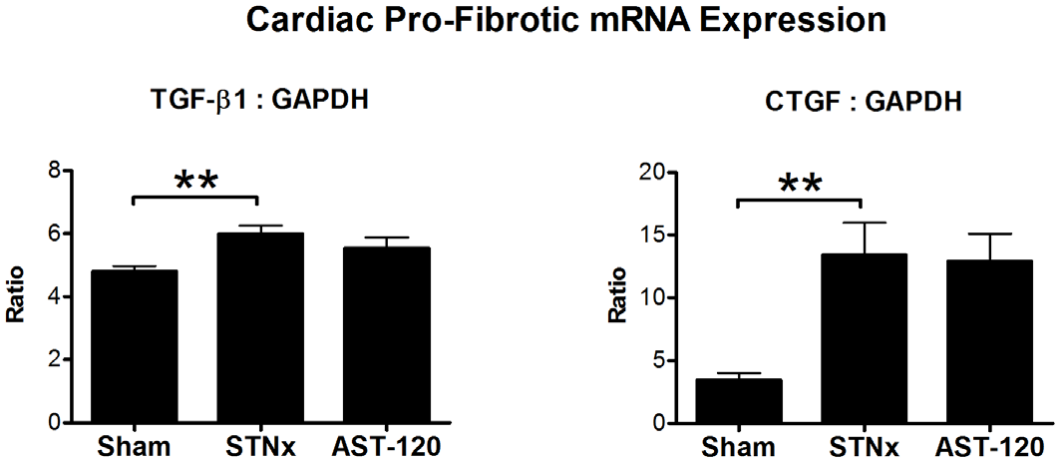
Increased cardiac TGF-β and CTGF mRNA expression in STNx animals is unchanged with AST-120 treatment at 12 **weeks post-surgery.** **p<0.01 vs Sham.

Cardiac TGF-β1 and CTGF mRNA expression was significantly increased in STNx + Vehicle compared to sham (p<0.01), these were unaffected by AST-120 treatment (Fig. 3).

**Figure 4:**
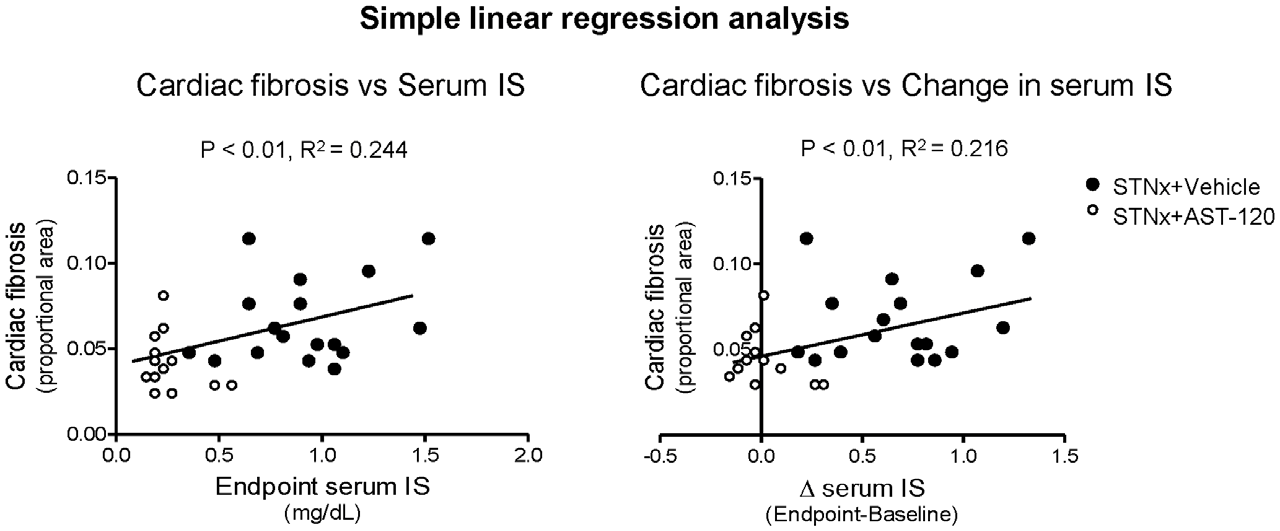
Cardiac fibrosis positively correlates with serum IS levels (left) and change in serum IS levels (right).

Degree of cardiac fibrosis was positively correlated with serum IS levels (endpoint and change after STNx surgery) (Fig. 4, p<0.01). On multivariate linear regression analysis, this was independent of tail-cuff BP, serum creatinine and 24-hour urine total protein.

#### Hypertrophy

**Figure 5:**
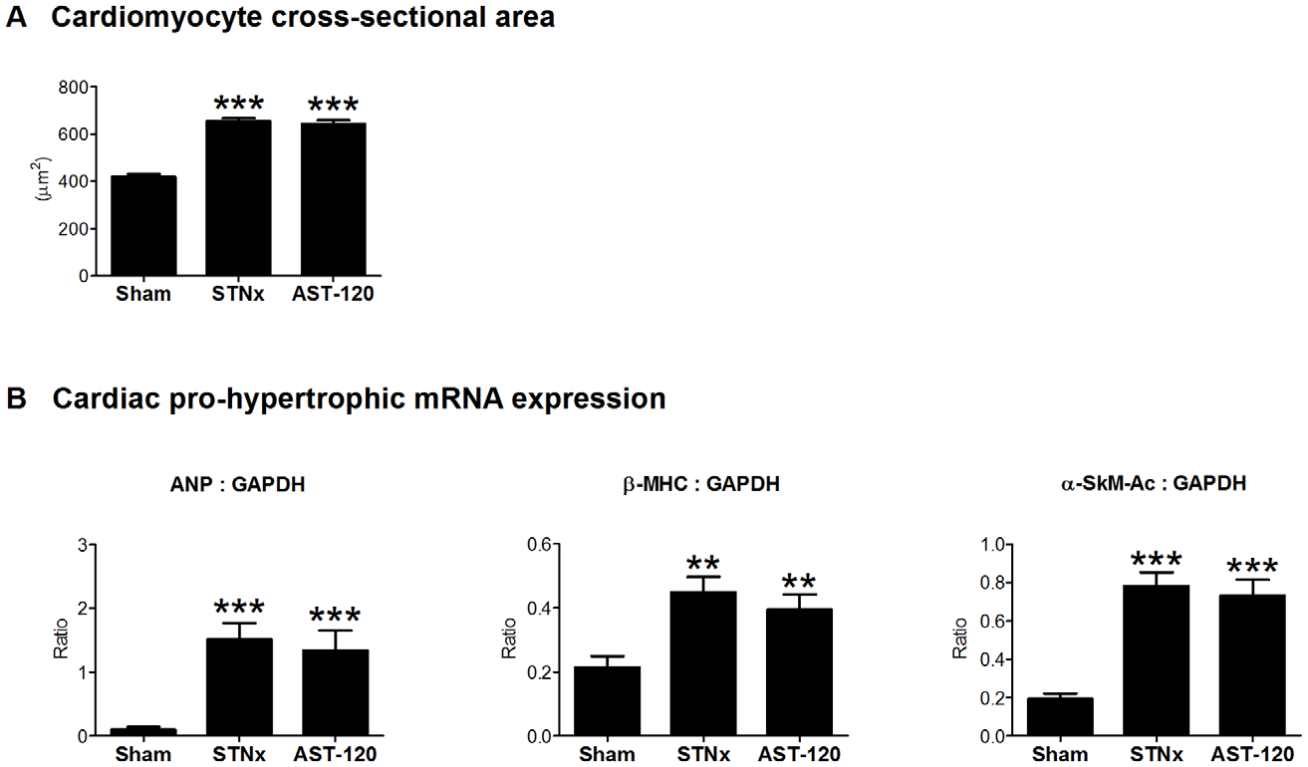
AST-120 shows no effects on cardiac hypertrophy as well as pro-hypertrophic mRNA expression in the STNx model. Compared to sham, STNx animals show a significant increase in cardiomyocyte cross-sectional area (A) and cardiac mRNA expression of ANP, β-MHC and α-SkM-Ac genes (B). There was no difference between untreated and AST-120-treated groups. **p<0.01, ***p<0.001 vs Sham.

LV cardiomyocyte cross-sectional area was significantly increased in STNx + Vehicle compared to sham animals (p<0.001). This was unaffected with AST-120 treatment (Fig. 5A).

Expression of ANP, β-MHC and α-SkM-Ac mRNA was increased in both STNx + Vehicle and STNx + AST-120 groups (Fig. 5B). AST-120 had no effect on pro-hypertrophic gene expression.

#### Pro-inflammatory cytokine mRNA expression

A non-significant increase in cardiac IL-6 mRNA expression was observed in STNx + Vehicle animals. Treatment with AST-120 non-significantly reduced expression levels of IL-6 (data not shown). There was no difference in cardiac TNF-α mRNA expression among groups. Cardiac IL-1β mRNA expression was expressed at levels too low to be detected with the same protocol used to examine the other genes.

## Discussion

In the present study, reductions in serum levels of the non-dialysable uremic toxin, indoxyl sulfate, by the oral adsorbent, AST-120, reduces the pathological cardiac fibrosis associated with chronic kidney disease. This amelioration of cardiac fibrosis with AST-120 occurred in the absence of any change in blood pressure.

CKD patients are more at risk of CV death than developing end-stage renal disease. [2] The most common causes of CV death are sudden cardiac death (SCD) and heart failure [6] which differs from the general population. Coronary artery disease is also commonly present in CKD patients but accounts for a much smaller proportion than sudden cardiac death. [19] Increased risk of SCD is associated with many factors such as cardiac structural changes or the so-called uremic cardiomyopathy, large volume and rapid electrolyte shifts during dialysis and derangements in autonomic function. [20] Myocardial fibrosis with accompanying hypertrophy, can mechanically impede electrical propagation which induces electrical instability leading to arrhythmias and SCD by re-entry mechanisms. [19] Noting that pathological findings contributing to CV diseases in the CKD population are not only limited to the heart but also present in blood vessels. For instance, arterial stiffness and aortic calcification are common vascular abnormalities correlated to left ventricular hypertrophy [21] and possibly coronary heart disease if affecting coronary arteries. [22].

Traditional CV risk factors such as hypertension, diabetes mellitus and hyperlipidemia have less impact in CKD patients than in the general population and are insufficient to explain the unacceptably high prevalence of CV mortality. [23] A number of non-traditional risk factors have been proposed as a contributor to the progression of CKD including uremic toxins, in particular the protein-bound subgroup. As previously mentioned, removal of protein-bound uremic toxins is limited by current conventional hemodialysis. Among these toxins, IS has extensively been studied with regard to its adverse renal effects. IS has been demonstrated to be implicated in increased renal oxidative stress, activation of renal pro-fibrotic gene expression and progressive tubulointerstitial fibrosis and glomerular sclerosis leading to further damage of the remaining nephrons. [24].

Given common modes of induction of fibrosis in organs such as heart and kidney we hypothesized that IS may also induce cardiac fibrosis (as well as hypertrophy). We have reported this in our rat cardiac cell culture studies. [18] The aim of the present study was to test the hypothesis that reduction of serum IS levels that accumulate in chronic kidney disease can prevent the cardiac fibrosis that occurs in response to IS, as we had previously demonstrated *in vitro*.

An oral carbonaceous adsorbent, AST-120, is well known as an IS-lowering agent. AST-120 blocks the conversion of the IS precursor, indole (a derivative of tryptophan), in the gut before it is converted to IS by sulfation (a conjugation reaction which is part of the liver detoxification system). A renoprotective effect of AST-120 has previously been observed in both the CKD patient setting as well as in pre-clinical models of renal dysfunction. Improved renal function, delayed progression of CKD and improved survival as well as a decrease in serum IS, urine IS and plasma TGF-β1 levels have been reported in non-dialysis CKD patients with AST-120 treatment. [25], [26], [27], [28] In a STNx animal model, administration of AST-120 prevents progressive glomerular sclerosis and renal cortical interstitial fibrosis. [16], [29] Increased renal TGF-β1 mRNA expression and NF-κB activation are induced by IS, both *in vitro* and *in vivo*, [17], [30] and these have been demonstrated *in vivo* to be suppressed by AST-120. [17], [29].

Recently, several clinical studies have reported beneficial cardiovascular and cardiorenal effects of AST-120. Pre-dialysis CKD patients who received AST-120 for more than 6 months had a significant reduction in LV mass. [31] Another study of pre-dialysis CKD patients showed significantly reduced arterial stiffness and carotid intima media thickness. [32] Cardiorenal protective effects of long-term (2 years) AST-120 treatment have been demonstrated in chronic heart failure patients with moderate CKD. These patients had an improvement in renal function, edema, ANP levels, cardiothoracic ratio, length of hospital stay and a reduced number of admissions after AST-120 treatment, compared to before treatment. [33] However, mechanisms of the cardioprotective effects of AST-120 have not been not well-explored.

In the present study, the STNx model consistently produces hypertension, fibrosis, hypertrophy and diastolic dysfunction that may precede development of systolic dysfunction. Levels of the uremic toxin, IS, are markedly elevated in this model, consistent with the observations made in clinical practice where the toxin is unable to be adequately dialyzed and therefore accumulates in a progressive manner. [34] Our finding that cardiac fibrosis was reduced by AST-120 is parallel to reductions of serum and urine IS levels. These changes occurred independently of changes in blood pressure, suggesting an important contribution of uremic toxins to the so-called uremic cardiomyopathy. A positive correlation between cardiac fibrosis and serum IS levels, independent of improvements in renal function (serum creatinine and 24-hour urine total protein) suggest that the reduction of cardiac fibrosis by AST-120 treatment is at least in part directly due to its IS lowering effect.

Mechanistic analysis of AST-120 reducing cardiac fibrosis showed a significant reduction in cardiac TGF-β and phospho-NFκB p65 protein expression. These findings are consistent with those previously reported in proximal tubular cells following AST-120 administration. [35] This study also demonstrated IS-induced phospho-NFκB p65 activation in cultured human proximal tubular cells. Inhibition of NFκB p65 using an antioxidant suppressed IS-induced TGF-β1 protein expression in uremic rats. The authors concluded that the adverse effects of IS on the proximal tubular cells may be mediated *via* reactive oxygen species-NF-κB-TGF-β1 pathway. The same pathway may play a role in IS-induced cardiac fibrosis in the present study.

NF-κB p65/p50 is a common dimer of the classical pathway of NF-κB activation, which plays a pivotal role in regulating both nuclear translocation and gene transcription. The anti-phospho-NF-κB p65 antibody used in the present study is specific for the phosphorylation of p65 on serine 536 located in C-terminal transcription activation domain. Phosphorylation of p65 at serine 536 is associated with nuclear translocation following activation. [36] Thus, the IS-induced NF- κB p65 (Ser536) phosphorylation is likely to represent the functionality of NF-κB. In addition, an increase in TGF- β protein expression observed in the present study suggests that TGF- β may be one of the target genes transcribed by an NF-κB in the IS-induced cardiac fibrosis pathway.

The anti-fibrotic effect of AST-120 on the kidney has not only been demonstrated in a severe CKD model of 5/6-STNx but also in the less severe 3/4-STNx [29], [37], combined unilateral nephrectomy and diabetic nephropathy [38] and diabetic nephropathy only models. [39] As previously mentioned, renal fibrosis is also a key characteristic finding in progressive CKD. This suggests that administration of AST-120 in earlier stages of CKD may prevent CKD progression and in turn prevent cardiovascular complications.

Systemic inflammatory activation is common and is a critical mechanism in the progression of both CKD [40] and HF. [41] However, the present study did not observe any significant changes in cardiac pro-inflammatory mRNA expression in both STNx + Vehicle and STNx + AST-120 groups. There was no change in the mRNA levels of TGF-β1 and CTGF while a reduction in the protein levels was observed in this study. This may be explained by a different pattern of gene compared to protein activation where by 16 weeks post-STNx gene activation had already peaked and then declined prior to the observed increase in protein expression.

Our group has investigated effects of protein-bound uremic toxins on cultured cardiac fibroblasts determined by ^3^H-proline incorporation. The results showed that indoxyl sulfate had the strongest pro-fibrotic effect while *p*-cresol had a weak effect and *m*-cresol and *m*- and *p*-cresyl sulfate had no pro-fibrotic effect. [42] IS has recently been demonstrated as the most promising biomarker of the effect of AST-120 in STNx rats among other problematic protein-bound uremic toxins (such as *p*-cresyl sulfate, hippuric acid, phenyl sulfate and 4-ethylphenyl sulfate) of which circulatory levels are reduced by AST-120. [43].

These findings are of considerable clinical significance given the large burden of cardiovascular morbidity and mortality associated with patients who have CKD. Given that AST-120 is available in a number of countries (and currently in Phase III studies in Europe and North America), these findings are also of therapeutic relevance given that circulating IS levels were reduced and cardiac fibrosis ameliorated with oral administration in the present study.

The present study also sought to determine mechanisms by which such amelioration of cardiac fibrosis in response to AST-120 might be occurring. We observed a reduction in TGF-β protein expression supporting this pathway as a key contributor to the reduction in pathological cardiac fibrosis in the uremic cardiomyopathy setting, *via* reduction of uremic toxins.

### Study limitiations

#### Study limitiations

In most patients with CKD, some form of renin angiotensin aldosterone system (RAAS) blockade would be prescribed as background therapy. However, only a partial, not complete, reversal of uremic cardiomyopathy is observed with ACE inhibitors in CKD patients on hemodialysis. [44] The present study did not include a RAAS blocker, either as comparator or in combination with AST-120, since we wished to first conduct a proof-of-concept study examining the contribution of uremic toxins to cardiac fibrosis, as well as the potential therapeutic implications of reducing of serum levels of IS. Such combination studies to RAAS blockers remain to be performed and will be of major clinical relevance.

We also did not observe any major evidence of amelioration of cardiac dysfunction observed in the sub-total nephrectomy setting by AST-120. Subtle changes in cardiac fibrosis with AST-120 treatment may not necessarily be accompanied by functional alterations at 12 weeks. This may be at least in part representative of the time it takes for pathological cardiac fibrosis to contribute to the subsequent dysfunction and conversely the time it may take for reduction in pathological cardiac fibrosis to contribute to reversal of the ventricular remodeling process. Unfortunately, the 5/6 subtotal nephrectomy model is extremely aggressive and longer term follow-up studies to test this hypothesis may be difficult to conduct due to the high mortality observed over a relatively short period of time.

Nevertheless, despite these considerations, the present study has demonstrated a clear beneficial effect on pathological cardiac fibrosis using a strategy to reduce serum indoxyl sulfate levels in rats with chronic kidney disease. Given the absence of changes in blood pressure these findings would suggest that uremic toxemia contributes directly to the cardiac fibrosis observed in this setting and that reduction in circulating levels of uremic toxins, specifically that of indoxyl sulfate, may be of therapeutic benefit with regard to the cardiac effects of CKD.

## Methods

### Study Design

Male Sprague-Dawley rats (220–250 g) underwent subtotal (5/6) nephrectomy (STNx). Briefly, animals were anesthetized with 1.0–2.0% isoflurane mixed with oxygen. An upper midline abdominal incision was performed. Left renal artery and its branches were exposed and two-third of the blood supply to the left kidney was blocked by ligation with a 4.0 silk suture. A surface area of tissue discoloration was re-examined to confirm 2/3 infarction. The right renal artery was then ligated and the right kidney removed and abdominal wall and skin sutured. When conscious, all animals received subcutaneous buprenorphine (0.01 mg/kg, s.c.) for analgesia. STNx animals were then randomized after a full recovery to receive either AST-120 (STNx + AST-120, n = 13) or no treatment (STNx + vehicle, n = 17) for 12 weeks. AST-120 (Kremezin®, Kureha Pharmaceuticals, Tokyo, Japan) was administrated post-operatively in the chow at 8% w/w. Sham operated rats (n = 12) were used as controls.

Serum IS level was measured at baseline, 8 weeks and 12 weeks; and urine IS at 8 and 12 weeks. Cardiac and renal function, including hemoglobin levels was assessed prior to sacrifice at 12 weeks. Tail-cuff blood pressure was measured in conscious rats at 4, 8 and 12 weeks post-STNx.

Tissues were assessed for pathological and molecular changes using histological methods, western blot analysis and real-time PCR.

The investigation conformed with the Guide for the Care and Use of Laboratory Animals published by the US National Institutes of Health (PHS Approved Animal Welfare Assurance no. A5587-01). All animal usage was approved by St Vincent’s Hospital’s Animal Ethics Committee (AEC) in accordance with National Health and Medical Research Council (NHMRC) guide for the care and use of laboratory animals (AEC no. 005/09).

### Cardiac Function Assessment – Echocardiography and Millar Catheterization

At the end of 12 weeks, echocardiography was performed in lightly anaesthetized animals (ketamine 40 mg/kg, xylazine 5 mg/kg, ip) using a Vivid 7 (GE Vingmed, Horten, Norway) echocardiography machine with a 10 MHz phased array probe. The procedure was performed as per published standard protocol and as routinely performed in our laboratory. [45].

Animals were anesthetized with pentobarbitone (30 mg/kg, i.p.) and intubated for cardiac catheterization procedures, as previously described. [46] Briefly, animals were ventilated and a 2F miniaturized combined catheter/micromanometer (Model SPR838 Millar instruments, Houston, TX) was inserted into the right common carotid artery to obtain aortic blood pressure and then advanced into the left ventricle to obtain left ventricular pressure–volume (PV) loops. PV loops were recorded at steady state and during transient preload reduction, achieved by occlusion of the inferior vena cava and portal vein with the ventilator turned off and animal apnoeic. The following validated parameters were assessed using Millar conductance data acquisition and analysis software PVAN 3.2: left ventricular end systolic pressure (LVESP), left ventricular end diastolic pressure (LVEDP), dP/dt_max_, dP/dt_min_, Tau (t Logistic and τ Weiss at steady state), and the slope of the preload recruitable stroke work (PRSW) relationship.

### Glomerular Filtration Rate (GFR)

One day prior to sacrifice, GFR was performed to measure kidney function. Briefly, animals were injected with 0.26 ml (i.v.) of the radioactive isotope, ^99^technetium-diethylene triamine penta-acetic acid (^99^Tc-DTPA) prepared at a rate of 37 MBq/ml (1mCi/mL). Animals were bled 43 minutes later and their plasma radioactivity measured and compared to the counts of the standard reference prepared at the time of injection. GFR/kg body weight was calculated as described in previous studies published by our group. [47].

### Biochemical Analysis

Serum was separated from blood by centrifugation at 3000 r.p.m. for 15 min, and samples were stored at −80°C. Urine samples were stored at −20°C until analysis. Serum and urine IS levels were measured by a high performance liquid chromatography-mass spectrometry (HPLC-MS). [48] Sample (10 µl) were analyzed with HPLC-MS by mobile phase using 5% tetrahydrofuran/0.1 M KH_2_PO_4_ (pH 6.5) at a flow rate of 1 ml/min, and fluorescence detection (excitation 295 nm and emission 390 nm.) [48] Serum creatinine, urine creatinine and urine total protein levels were measured using Cobas Integra® 400 Plus Bioanalyzer (Roche, Indianapolis, IN) as per manufacturers’ instructions. Creatinine clearance was calculated using the following formula:
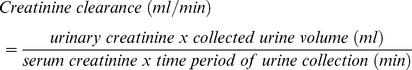



### Histological Analysis

Hearts were removed after catheterization measurements and weighed, fixed in 10% neutral buffered formalin and then processed for histopathology. Paraffin-embedded sections (4 µm) were prepared for histological staining.

#### Cardiac interstitial fibrosis

Cardiac sections, stained with picrosirius red for matrix deposition, [49] were scanned (Aperio, Aperio Technologies Inc., Vista, CA) for analysis. Picrosirius red-stained matrix deposition from the whole LV myocardium, excluding perivascular fibrosis was selected for its intensity, and the proportional area was calculated using a preset algorithm for picrosirius red stain intensity. [50].

### Cardiomyocyte Cross-sectional Area

Fifty LV cardiomyocytes with equal-sized nuclei were randomly selected for analysis of cross-sectional area from pre-scanned images. Cell surface areas were calculated by measuring circumferential length of the myocyte using Aperio ScanScope (Aperio, Aperio Technologies Inc., Vista, CA). Measurements from each animal were averaged and data expressed as mean ± SEM for each group.

### Western Blot Analysis

Supernatant from homogenized LV cardiac tissue (30 mg) was collected and protein concentrations measured by the Bradford assay (Bio-Rad, Hercules, CA, USA). Equal amounts of protein (30 µg) were separated by 10% sodium dodecyl sulfate–polyacrylamide gel electrophoresis, and electrophoretically transferred to nitrocellulose membranes (Amersham Biosciences). [51] Western blot analysis was performed as per manufacturer protocol with specific antibodies (TGF-β, phospho-p44/42, p44/42, phospho-p38, p38, phospho-nuclear factor kappa B (NFκB) p65 (Ser536), NF-κB p65 antibodies– Cell Signaling Technology, Beverly, MA, USA; pan-actin antibody – NeoMarkers, Fremont, CA) and then visualized by enhanced chemiluminescence reagents (Thermo Scientific). Band intensity was analyzed using ImageJ software (NCBI). [18] Pan-actin and total-proteins were used as endogenous controls to correct for non-phosphorylated protein and the corresponding phosphorylated-protein expression, respectively.

### Quantitative mRNA Expression in Left Ventricular Tissue

Total RNA was extracted from 30 mg of cardiac tissue using the RNAqueous™ kit (Ambion, Austin, TX). RNA was reverse transcribed to cDNA and triplicate cDNA aliquots were amplified using either sequence-specific primers (Geneworks, Adelaide, SA, Australia) with SYBR Green detection (for pro-fibrotic and pro-inflammatory cytokine genes; Applied Biosystems) or premixed primer-probe (for pro-hypertrophic genes and GAPDH; Applied Biosystems) using an ABI prism 7900 HT Sequence Detection System (Applied Biosystems). Real-time polymerase chain reaction (PCR) was used to quantify pro-fibrotic [transforming growth factor-beta 1(TGF-β1), connective tissue growth factor (CTGF)], pro-hypertrophic [atrial natriuretic peptide (ANP), beta-myosin heavy chain (β-MHC), alpha-skeletal muscle actin (α-SkM-Ac)] and pro-inflammatory cytokine (TNF-α, IL-6, IL-1β) gene expression. The primer pairs were designed using Primer Express 2.0 software (Applied Biosystems) based on published sequences (http://www.ncbi.nlm.nih.gov). The forward primer of TGF-β1 was 5′-CCA GCC GCG GGA CTC T-3′, and the reverse primer was 5′-TTC CGT TTC ACC AGC TCC AT-3′; and the forward primer of CTGF was 5′-GCG GCG AGT CCT TCC AA-3′, and the reverse primer was 5′-CCA CGG CCC CAT CCA-3′. GAPDH, used as the endogenous control to correct for the expression of each gene, ANP, β-MHC and α-SkM-Ac probe and primers were obtained from Applied Biosystems Assays on Demand.

### Statistical Analysis

Data are presented as means ± SEM. One-way ANOVA with Bonferroni’s multiple comparison test or Kruskal-Wallis test with Dunn’s multiple comparison test were used for comparisons among all groups for parametric and non-parametric data, respectively. For comparisons between 2 groups, unpaired Student t-test was used for parametric data and Mann Whitney test for non-parametric data. Statistical analyses were performed by using GraphPad Prism software version 5 and multiple linear regression analysis by SPSS. A two-tailed p-value of less than 0.05 was considered statistically significant.

## References

[1] United States Renal Data Systems. USRDS 2007 Annual Data Report:Atlas of End-Stage Renal Disease in the United States.. Bethesda, MD: National Institute of Health, National Institute of Diabetes and Digestive and Kidney Diseases.

[2] Sarnak MJ, Levey AS, Schoolwerth AC, Coresh J, Culleton B, et al (2003). Kidney disease as a risk factor for development of cardiovascular disease: a statement from the American Heart Association Councils on Kidney in Cardiovascular Disease, High Blood Pressure Research, Clinical Cardiology, and Epidemiology and Prevention.. Circulation.

[3] Amann K, Buzello M, Simonaviciene A, Miltenberger-Miltenyi G, Koch A (2000). Capillary/myocyte mismatch in the heart in renal failure–a role for erythropoietin?. Nephrol Dial Transplant.

[4] Vlahakos DV, Hahalis G, Vassilakos P, Marathias KP, Geroulanos S (1997). Relationship between left ventricular hypertrophy and plasma renin activity in chronic hemodialysis patients.. J Am Soc Nephrol.

[5] Zoccali C, Mallamaci F, Tripepi G, Parlongo S, Cutrupi S (2002). Norepinephrine and concentric hypertrophy in patients with end-stage renal disease.. Hypertension.

[6] Gross ML, Ritz E (2008). Hypertrophy and fibrosis in the cardiomyopathy of uremia–beyond coronary heart disease.. Semin Dial.

[7] Go AS, Yang J, Ackerson LM, Lepper K, Robbins S (2006). Hemoglobin level, chronic kidney disease, and the risks of death and hospitalization in adults with chronic heart failure: the Anemia in Chronic Heart Failure: Outcomes and Resource Utilization (ANCHOR) Study.. Circulation.

[8] Upadhyay A, Larson MG, Guo CY, Vasan RS, Lipinska I (2011). Inflammation, kidney function and albuminuria in the Framingham Offspring cohort.. Nephrol Dial Transplant.

[9] Zoccali C, Bode-Boger S, Mallamaci F, Benedetto F, Tripepi G (2001). Plasma concentration of asymmetrical dimethylarginine and mortality in patients with end-stage renal disease: a prospective study.. Lancet.

[10] Hampl H, Hennig L, Rosenberger C, Amirkhalily M, Gogoll L (2005). Effects of optimized heart failure therapy and anemia correction with epoetin beta on left ventricular mass in hemodialysis patients.. Am J Nephrol.

[11] Tornig J, Amann K, Ritz E, Nichols C, Zeier M (1996). Arteriolar wall thickening, capillary rarefaction and interstitial fibrosis in the heart of rats with renal failure:the effects of ramipril, nifedipine and moxonidine.. J Am Soc Nephrol.

[12] Cice G, Ferrara L, D’Andrea A, D’Isa S, Di Benedetto A (2003). Carvedilol increases two-year survivalin dialysis patients with dilated cardiomyopathy: a prospective, placebo-controlled trial.. J Am Coll Cardiol.

[13] Cannella G, Paoletti E, Delfino R, Peloso G, Rolla D (1997). Prolonged therapy with ACE inhibitors induces a regression of left ventricular hypertrophy of dialyzed uremic patients independently from hypotensive effects.. Am J Kidney Dis.

[14] Wikoff WR, Anfora AT, Liu J, Schultz PG, Lesley SA (2009). Metabolomics analysis reveals large effects of gut microflora on mammalian blood metabolites.. Proc Natl Acad Sci U S A.

[15] Stanfel LA, Gulyassy PF, Jarrard EA (1986). Determination of indoxyl sulfate in plasma of patients with renal failure by use of ion-pairing liquid chromatography.. Clin Chem.

[16] Niwa T, Ise M (1994). Indoxyl sulfate, a circulating uremic toxin, stimulates the progression of glomerular sclerosis.. J Lab Clin Med.

[17] Miyazaki T, Aoyama I, Ise M, Seo H, Niwa T (2000). An oral sorbent reduces overload of indoxyl sulphate and gene expression of TGF-beta1 in uraemic rat kidneys.. Nephrol Dial Transplant.

[18] Lekawanvijit S, Adrahtas A, Kelly DJ, Kompa AR, Wang BH (2010). Does indoxyl sulfate, a uraemic toxin, have direct effects on cardiac fibroblasts and myocytes?. Eur Heart J.

[19] Glassock RJ, Pecoits-Filho R, Barberato SH (2009). Left ventricular mass in chronic kidney disease and ESRD.. Clin J Am Soc Nephrol.

[20] Herzog CA, Mangrum JM, Passman R (2008). Sudden cardiac death and dialysis patients.. Semin Dial.

[21] Nitta K, Akiba T, Uchida K, Otsubo S, Otsubo Y (2004). Left ventricular hypertrophy is associated with arterial stiffness and vascular calcification in hemodialysis patients.. Hypertens Res.

[22] Nitta K, Akiba T, Suzuki K, Uchida K, Ogawa T (2004). Assessment of coronary artery calcification in hemodialysis patients using multi-detector spiral CT scan.. Hypertens Res.

[23] Foley RN, Parfrey PS, Sarnak MJ (1998). Epidemiology of cardiovascular disease in chronic renal disease.. J Am Soc Nephrol.

[24] Niwa T (2010). Uremic toxicity of indoxyl sulfate.. Nagoya J Med Sci.

[25] Maeda K, Hamada C, Hayashi T, Shou I, Wakabayashi M (2009). Long-term effects of the oral adsorbent, AST-120, in patients with chronic renal failure.. J Int Med Res.

[26] Niwa T, Nomura T, Sugiyama S, Miyazaki T, Tsukushi S (1997). The protein metabolite hypothesis, a model for the progression of renal failure: an oral adsorbent lowers indoxyl sulfate levels in undialyzed uremic patients.. Kidney Int.

[27] Ueda H, Shibahara N, Takagi S, Inoue T, Katsuoka Y (2008). AST-120 treatment in pre-dialysis period affects the prognosis in patients on hemodialysis.. Ren Fail.

[28] Iida S, Kohno K, Yoshimura J, Ueda S, Usui M (2006). Carbonic-adsorbent AST-120 reduces overload of indoxyl sulfate and the plasma level of TGF-beta1 in patients with chronic renal failure.. Clin Exp Nephrol.

[29] Tamada S, Asai T, Kuwabara N, Iwai T, Uchida J (2006). Molecular mechanisms and therapeutic strategies of chronic renal injury: the role of nuclear factor kappaB activation in the development of renal fibrosis.. J Pharmacol Sci.

[30] Motojima M, Hosokawa A, Yamato H, Muraki T, Yoshioka T (2003). Uremic toxins of organic anions up-regulate PAI-1 expression by induction of NF-kappaB and free radical in proximal tubular cells.. Kidney Int.

[31] Nakai K, Fujii H, Kono K, Goto S, Fukagawa M (2011). Effects of AST-120 on left ventricular mass in predialysis patients.. Am J Nephrol.

[32] Nakamura T, Kawagoe Y, Matsuda T, Ueda Y, Shimada N (2004). Oral ADSORBENT AST-120 decreases carotid intima-media thickness and arterial stiffness in patients with chronic renal failure.. Kidney Blood Press Res.

[33] Shibahara H, Shibahara N (2010). Cardiorenal protective effect of the oral uremic toxin absorbent AST-120 in chronic heart disease patients with moderate CKD.. J Nephrol.

[34] Barreto FC, Barreto DV, Liabeuf S, Meert N, Glorieux G (2009). Serum indoxyl sulfate is associated with vascular disease and mortality in chronic kidney disease patients.. Clin J Am Soc Nephrol.

[35] Shimizu H, Bolati D, Adijiang A, Muteliefu G, Enomoto A (2011). NF-kappaB plays an important role in indoxyl sulfate-induced cellular senescence, fibrotic gene expression, and inhibition of proliferation in proximal tubular cells.. Am J Physiol Cell Physiol.

[36] Sasaki CY, Barberi TJ, Ghosh P, Longo DL (2005). Phosphorylation of RelA/p65 on serine 536 defines an I{kappa}B{alpha}-independent NF-{kappa}B pathway.. J Biol Chem.

[37] Kobayashi N, Maeda A, Horikoshi S, Shirato I, Tomino Y (2002). Effects of oral adsorbent AST-120 (Kremezin) on renal function and glomerular injury in early-stage renal failure of subtotal nephrectomized rats.. Nephron.

[38] Aoyama I, Shimokata K, Niwa T (2002). An oral adsorbent downregulates renal expression of genes that promote interstitial inflammation and fibrosis in diabetic rats.. Nephron.

[39] Aoyama I, Shimokata K, Niwa T (2000). Oral adsorbent AST-120 ameliorates interstitial fibrosis and transforming growth factor-beta(1) expression in spontaneously diabetic (OLETF) rats.. Am J Nephrol.

[40] Stenvinkel P (2006). Inflammation in end-stage renal disease: the hidden enemy.. Nephrology (Carlton).

[41] Mann DL (2002). Inflammatory mediators and the failing heart: past, present, and the foreseeable future.. Circ Res.

[42] Iyngkaran P, Tuck KL, Ma J, Ho P, Lekawanvijit S (2009). Do all protein bound renal toxins exert physiological effects on cardiac cells?. Heart Lung and Circulation.

[43] Kikuchi K, Itoh Y, Tateoka R, Ezawa A, Murakami K (2010). Metabolomic search for uremic toxins as indicators of the effect of an oral sorbent AST-120 by liquid chromatography/tandem mass spectrometry.. J Chromatogr B Analyt Technol Biomed Life Sci.

[44] London GM, Pannier B, Guerin AP, Blacher J, Marchais SJ (2001). Alterations of left ventricular hypertrophy in and survival of patients receiving hemodialysis: follow-up of an interventional study.. J Am Soc Nephrol.

[45] Phrommintikul A, Tran L, Kompa A, Wang B, Adrahtas A (2008). Effects of a Rho kinase inhibitor on pressure overload induced cardiac hypertrophy and associated diastolic dysfunction.. Am J Physiol Heart Circ Physiol.

[46] Kompa AR, Wang BH, Phrommintikul A, Ho PY, Kelly DJ (2010). Chronic urotensin II receptor antagonist treatment does not alter hypertrophy or fibrosis in a rat model of pressure-overload hypertrophy.. Peptides.

[47] Kelly DJ, Wilkinson-Berka JL, Allen TJ, Cooper ME, Skinner SL (1998). A new model of diabetic nephropathy with progressive renal impairment in the transgenic (mRen-2)27 rat (TGR).. Kidney Int.

[48] Owada S, Goto S, Bannai K, Hayashi H, Nishijima F (2008). Indoxyl sulfate reduces superoxide scavenging activity in the kidneys of normal and uremic rats.. Am J Nephrol.

[49] Tran L, Kompa AR, Kemp W, Phrommintikul A, Wang BH (2010). Chronic urotensin-II infusion induces diastolic dysfunction and enhances collagen production in rats.. Am J Physiol Heart Circ Physiol.

[50] Campbell DJ, Somaratne JB, Jenkins AJ, Prior DL, Yii M (2011). Differences in myocardial structure and coronary microvasculature between men and women with coronary artery disease.. Hypertension.

[51] Kompa AR, See F, Lewis DA, Adrahtas A, Cantwell DM (2008). Long-term but not short-term p38 mitogen-activated protein kinase inhibition improves cardiac function and reduces cardiac remodeling post-myocardial infarction.. J Pharmacol Exp Ther.

